# Nitric Oxide Dysregulation in Platelets from Patients with Advanced Huntington Disease

**DOI:** 10.1371/journal.pone.0089745

**Published:** 2014-02-25

**Authors:** Albino Carrizzo, Alba Di Pardo, Vittorio Maglione, Antonio Damato, Enrico Amico, Luigi Formisano, Carmine Vecchione, Ferdinando Squitieri

**Affiliations:** 1 IRCCS Neuromed, Pozzilli (IS), Italy; 2 Department of Science and Technology, University of Sannio, Benevento, Italy; 3 Department of Medicine and Surgery, University of Salerno, Salerno, Italy; Universidade de São Paulo, Brazil

## Abstract

Nitric oxide (NO) is a biologically active inorganic molecule involved in the regulation of many physiological processes, such as control of blood flow, platelet adhesion, endocrine function, neurotransmission and neuromodulation. In the present study, for the first time, we investigated the modulation of NO signaling in platelets of HD patients. We recruited 55 patients with manifest HD and 28 gender- and age-matched healthy controls. Our data demonstrated that NO-mediated vasorelaxation, when evoked by supernatant from insulin-stimulated HD platelets, gradually worsens along disease course. The defective vasorelaxation seems to stem from a faulty release of NO from platelets of HD patients and, it is associated with impairment of eNOS phosphorylation (Ser^1177^) and activity. This study provides important insights about NO metabolism in HD and raises the hypothesis that the decrease of NO in platelets of HD individuals could be a good tool for monitoring advanced stages of the disease.

## Introduction

Huntington disease (HD), a dominantly transmitted neurodegenerative disorder, is characterized by the progressive striatal and cortical neurodegeneration associated with motor, cognitive and behavioral disturbances [1]. The disease-causing mutation is an expansion of a CAG trinucleotide repeat (>36 repeats) encoding a polyglutamine (polyQ) stretch in N-terminal region of huntingtin (Htt), a ubiquitous protein whose function is still unclear. Elongated polyQ stretch endows mutant Htt (mHtt) with toxic properties and results in the development of a broad array of cell dysfunctions [2]. Although the disease has traditionally been described as a disorder purely of the brain, emerging evidence indicates that abnormalities outside the central nervous system (CNS) are commonly found in HD [3]. However, whether a correlation exists between central pathology and peripheral defects is still poorly understood.

Mutant Htt has been widely described to be expressed either in central or in peripheral tissues and to exert its toxic effect in both neuronal and non-neuronal cells with similar mechanisms [4].

Previous studies highlighted the ability of mHtt to interfere with a number of molecular mechanisms in peripheral tissues, which have been suggested to virtually reflect central dysfunctions in HD and potentially useful to better understand some genetic and biochemical aspects of the disease [5], [6].

Among all the several dysfunctions, the dysregulation of nitric oxide (NO)/NO synthase (NOS) pathway is suggested to potentially represent a critical contributor to HD pathology [7]. NO, known as an important signaling molecule, normally acts in many tissues and regulates a diverse range of physiological and cellular processes such as control of blood flow, platelet adhesion, neurotrasmission and neuromodulation [8]. Production of NO is mediated by different NOS isoforms in both CNS and peripheral tissues [9]. Human platelets, that normally express the endothelial form of NOS (eNOS), whose activity is regulated by phosphorylation at different Serine/Threonine residues [10], represent an important source of peripheral NO. Under pathological condition, production of NO can be either protective or toxic, depending on the stage of the disease, the isoforms of NOS involved and, the initial pathological event [7]. To date, many are the studies that described NO as a potential key mediator of neurodegeneration [11], [12].

NO dysfunction in CNS has been previously demonstrated to be involved in different processes leading to progressive striatal damage and to abnormal cerebral blood flow (CBF) in both HD experimental models and patients [7], [13]. However, there is no actual evidence proving NO abnormalities in peripheral blood cells.

In this study, we carried out experiments on platelets, which represent a validated peripheral model for testing potential impairment of NO regulation, obtained from patients with manifest HD. Our data highlighted defective NO metabolism in the advanced stages of HD and, for the first time, reveled a possible stage-dependent dysregulation of peripheral eNOS signaling in the disease.

## Methods

### Subjects

A total of 55 HD patients (11 stage I, 17 stage II, 16 stage III and 11 stage IV), and 28 gender- and age-matched healthy controls were recruited in this study. Control group was divided in two subgroups consisting of a younger control group with a mean age of 43.8±7.4 and an older control group with a mean age of 59.7±3.4 in order to match them with early (stage I and II) and late (stage III and IV) HD patients, respectively. Classification of control subjects in young and old groups has been performed in a manner similar to that described previously [14]. Subjects’ demographic, clinical and genetic characteristics of both controls and HD patients are reported in Table 1. All the subjects with suspect of cardiovascular, psychiatric or neurodegenerative disorders other than HD, were excluded from this study. Most patients were taking benzodiazepines; some of the patients in stage III-IV were receiving low doses of atypical neuroleptics (olanzapine, 2.5–10 mg; risperidone, 1–3mg or tetrabenazine, 12.5–25 mg). None of the patients were taking medication for any cardiovascular diseases. Clinical examinations were conducted using the Unified Huntington’s Disease Rating Scale (UHDRS) to measure motor, cognitive, behavioral and general function (Huntington Study Group, 1996) [15]. The disease stage was calculated according to the Total Functional Capacity (TFC) score [16].

**Table 1:** Demographic and clinical data of healthy controls and HD patients.

			Huntington disease stagesEarly Late			
	Young Controls	Old Controls	I	II	III	IV
**Subjects**	19	9	11	17	16	11
**Male/Female**	9/10	5/4	5/6	9/8	9/6	5/6
**Age (years)**	43.8±7.4	59.7±3.4	46.9±10.2	50.7±13.5	54.2±11.2	61.5±8.2
**TFC (score)**	-	-	11.5±1	7.8±0.7	4.1±1.3	1.6±0.5
**CAG repeats**	-	-	43.2±1.5	45.6±6.3	44.7 ± 3.4	42.3 ± 2.1

Values are given as mean ± s.d.; TFC: Total Functional Capacity, score 13-0.

### Ethics Statement

All HD patients revealed a CAG repeat expansion mutation, and all of them, as well as control individuals, were requested to sign an informed consent before study recruitment. All human experiments were performed in accordance with the Declaration of Helsinki and after approval from the local Ethical Committee of Istituto Neurologico Mediterraneo IRCCS Neuromed [17].

### Platelet isolation

Twenty-five millilitres of blood were collected into acid citrate dextrose (ACD: 85 mmol/L sodium citrate, 65 mmol/L citric acid, and 125 mmol/L dextrose; 2,5 ml ACD:25 ml of blood) and platelet-rich plasma was obtained by centrifugation at 130 *g* for 20 minutes. The resultant platelet-rich plasma (PRP) was used as source of platelets. Platelets pellet was obtained by centrifugation of PRP at 900 *g* for 7 minutes, and resuspended in Tyrode’s solution (132 mmol/L NaCl, 4 mmol/L KCl, 1.6 mmol/L CaCl_2_, 0.98 MgCl_2_, 23.8 mmol/L NaHCO_3_, 0.36 mmol/L NaH_2_PO_4_, 10 mmol/L glucose, 0.05 mmol/L Ca-Titriplex, and gassed with 95% O_2_, 5% CO_2_ and pH 7.4 at 37°C). After a further centrifugation step (900 *g*, 4 minutes), washed platelets were re-suspended in the same solution, allowed to equilibrate for 10 minutes at 37°C and then stimulated with insulin (10 µmol/L) for 10 minutes. Some experiments were performed in platelet pre-treated with N^G^-nitro-L-arginine methyl ester (L-NAME) (300 µmol/L, 30 minutes) or with 1 mmol/L 4-hydroxytempo (TEMPOL), a membrane-permeable superoxide dismutase mimetic, for 30 minutes. After stimulation, the platelet suspension was centrifuged for 2 minutes at 900 *g* and, increasing doses of supernatant (0.1-0.2-0.4-0.8 ml) was added to phenyleprine-precontracted arteries mounted in an organ chamber (final volume, 15 ml). Total number and purity of platelets for each preparation was assessed by flow cytometry. Total protein from each preparation was also determined. Similar number of platelets (186±15×10^6^/mL) between controls and HD patients was estimated.

### Isolated vessel studies

Studies of vascular reactivity were performed on isolated vessels from C57BL6/N mice. Four ring segments (3 mm width) of thoracic aorta from each mouse were mounted between stainless steel triangles in a water-jacketed organ bath (37°C) for measurement of tension development as previously described [18], [19]. Preliminary experiments demonstrated that the optimal resting tension for development of active contraction was 1 g. Vessels were gradually stretched over one-hour period to this tension. The presence of functional endothelium and smooth muscle layer were assessed in all preparations by the ability respectively of acetylcholine and nitrogliceryne (10^−9^ to 10^−5 ^mol/L) to induce the relaxation of vessels precontracted with phenylephrine (10^−9^ to 10^−6 ^mol/L) to obtain a similar level of precontraction in each ring (80% of initial KCl-induced contraction). Responses to vasoconstrictors were examined at this resting tension and related to maximal vasoconstriction elicited by depolarization with 80 mmol/L KCl. Responses to vasodilator supernatants obtained from stimulation of platelets were examined after achieving a preconstricted tone with increasing doses of phenylephrine (10^−9^ to 10^−6 ^mol/L) to obtain a similar level of precontraction in each ring (80% of initial KCl-induced contraction).

### Immunoblotting

After isolation, platelets were solubilized in lysis buffer containing: 20 mmol/L Tris-HCl, 150 mmol/L NaCl, 20 mmol/L NaF, 2 mmol/L sodium orthovanadate, 1% Nonidet, 100 µg/ml leupeptin, 100 µg/ml aprotinin, and 1 mmol/L phenylmethylsulfonyl fluoride. Then, samples were left on ice for 30 minutes and centrifuged at 10621 *g* for 20 minutes, and the supernatants were used to perform immunoblot analysis. Total protein levels were determined using the Bradford method. 50 µg proteins were resolved on 8% SDS-PAGE, transferred to a nitrocellulose membrane as previously described [20] and immunoblotted with anti-phospho-eNOS S1177 (Cell Signaling, rabbit polyclonal antibody 1:800); anti-total-eNOS (Cell Signaling, mouse mAb 1:1000) and β-actin (Cell Signaling, mouse mAb 1∶2000). HRP-conjugated secondary antibodies were used at 1∶3000 dilution (Bio-Rad Laboratories). Protein bands were detected by ECL Prime (Amersham Biosciences) and quantitated with Quantity One software (Bio-Rad Laboratories).

### Determination of platelet Nitric Oxide Synthase activity

Endothelial NOS activity was determined on platelet lysates according to the protocol published by Radomski et al. (1993) [21]. Briefly, the enzymatic activity was determined by measuring the conversion of *L*-[^14^C]arginine to *L*-[^14^C]citrulline. The dependence of *L*-arginine conversion on Ca_2_^+^-dependent (constitutive) NOS was confirmed in presence of ethyleneglycol bis-(2-aminoethyl ether)-*N,N,N*-tetraacetic acid (EGTA), and of N(G)-mono-methyl-*L*-arginine (L-NMMA), an inhibitor of this enzyme. The isoform specificity of this analysis is demonstrated through the use of the calcium chelator EGTA, which allows us to fractionate calcium-independent (iNOS) activity versus total NOS activity (inhibited by L-NMMA). Assay sensitivity has been shown to be just less than 1 pmol/min/mg protein for the detection of *L*-[^14^C]arginine.

### Measurement of NO production in platelets

Isolated platelets were centrifuged at 1500 *g* for 10 minutes and platelets were washed twice with a 6∶1 mixture of Hank’s balanced salt solution (HBSS) and ACD. Platelet suspensions (100 µL) were incubated with 1 µmol/L diaminodifluoroscein diacetate (DAF-FM DA), a photo-stable NO fluorescent indicator, in HBSS at room temperature for 30 minutes and stimulated with 10 µmol/L insulin. After incubation, fluorescence emission spectra were recorded in a 495-515 nm range in a quartz cuvette as described elsewhere [22].

### Statistical analysis

One-way ANOVA followed by Turkey post-test was used to analyze protein levels, eNOS activity and DAF fluorescence measurement. Two-way ANOVA followed by Bonferroni post-test was used for the analysis of vascular reactivity studies in HD patients and controls. All data are presented as mean ± SD, except in the dose-response curve studies in which are expressed as mean ± SEM. A “*p*” value of less than 0.05 was considered statistically significant. F value (F) and degree of freedom (df) were also reported for each experiment. All statistical analyses were conducted with Prism statistical software.

## Results

### Vasorelaxation evoked by supernatant from insulin-stimulated platelets gradually alters along disease course

**Figure 1:**
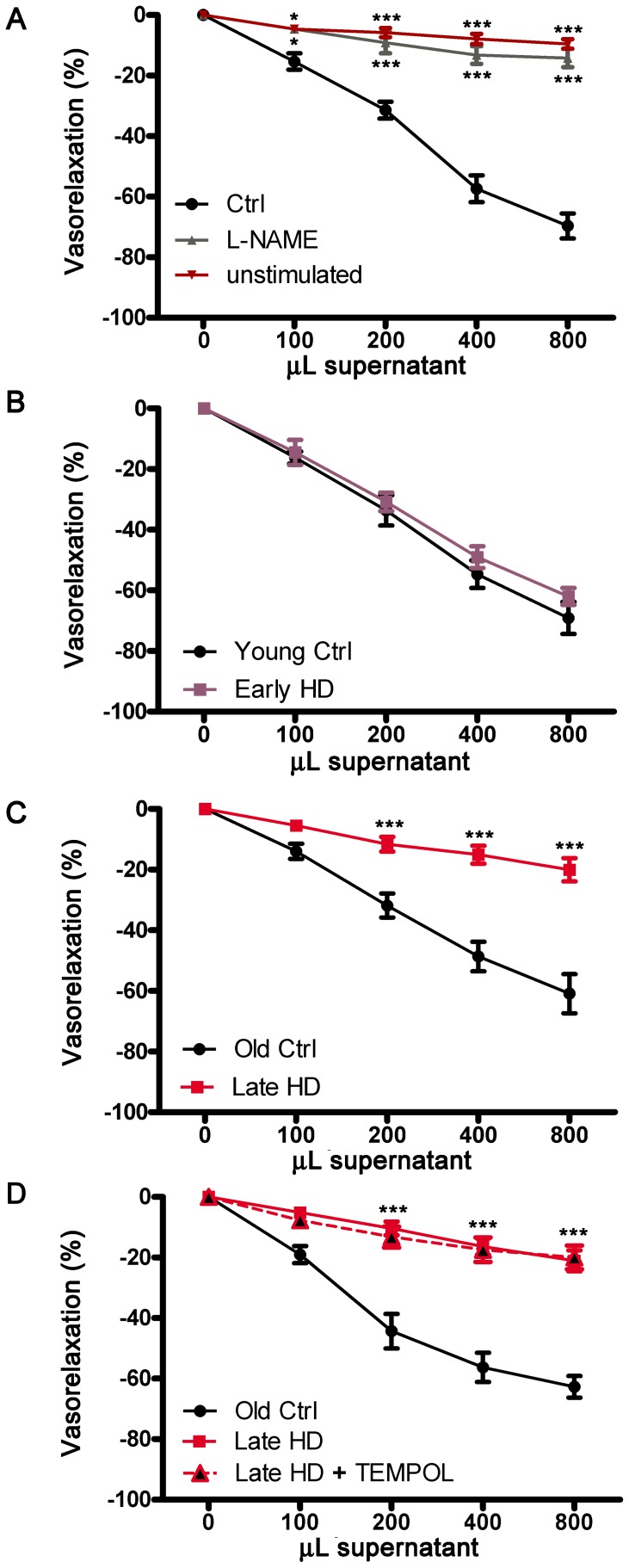
Vasorelaxant response to supernatants derived from insulin-stimulated platelets of HD patients. (**A**) Dose–response curves of phenylephrine-precontracted aorta rings to supernatants derived from insulin-stimulated and unstimulated platelets isolated from control subjects untreated and pre-treated with L-NAME. * indicates statistical significance of either Ctrls vs L-NAME-treated Ctrls or vs unstimulated samples. Ctrl n = 4; L-NAME-treated Ctrls n = 4; Unstimulated n = 4. (**B**) Dose–response curves of phenylephrine-precontracted aorta rings to supernatants derived from insulin-stimulated platelets isolated from early HD patients and young control subjects. Young Ctrl n = 13; Early HD n = 8. (**C**) Dose–response curves of phenylephrine-precontracted aorta rings to supernatants derived from insulin-stimulated platelets isolated from late HD patients and old control subjects. * indicates statistical significance of old Ctrl vs late HD. Old Ctrl n = 6; Late HD n = 7. (**D**) Dose–response curves of phenylephrine-precontracted aorta rings to supernatants derived from platelets isolated from Old control subjects and late HD patients untreated and pre-treated with TEMPOL before insulin stimulation. Old Ctrl n = 3; Late HD plus Tiron n = 4. * indicates statistical significance of old Ctrl vs late HD stages treated and untreated with TEMPOL. Values are shown as mean± SEM. *, *p*<0.05; **, *p* <0.001; ***, *p* <0.0001. **(**Two-way ANOVA followed Bonferroni post-test**).**

The supernatant from insulin-stimulated human platelets evoked a rapid dose-dependent relaxation of mice aorta rings and was abolished by NOS inhibitor, L-NAME (Figure 1A), clearly demonstrating the involvement of NO signalling in supernatant vascular action. Interestingly, our data demonstrate that the vasorelaxant effect, evoked from platelet supernatants, was markedly reduced in late stage HD patients when compared to either early HD patients or control subjects (Figure 1B). No differences were observed between early HD patients and age-matched control individuals (Figure 1B). Vasorelaxation did not differ between the two control groups, excluding, therefore, any possible aging process-related effects (Figure S1 in File S1).

Next, to elucidate the putative cause of aberrant NO pathway, the potential implication of oxidative stress was explored by pre-incubating platelets from late stage HD patients with the antioxidant agent, 4-hydroxytempo. Our data indicate that the compound failed to improve the supernatant vascular effect observed in late HD patients (Figure 1C), excluding the involvement of oxidative stress in such dysfunction.

### eNOS-phosphorylation is impaired in HD

**Figure 2:**
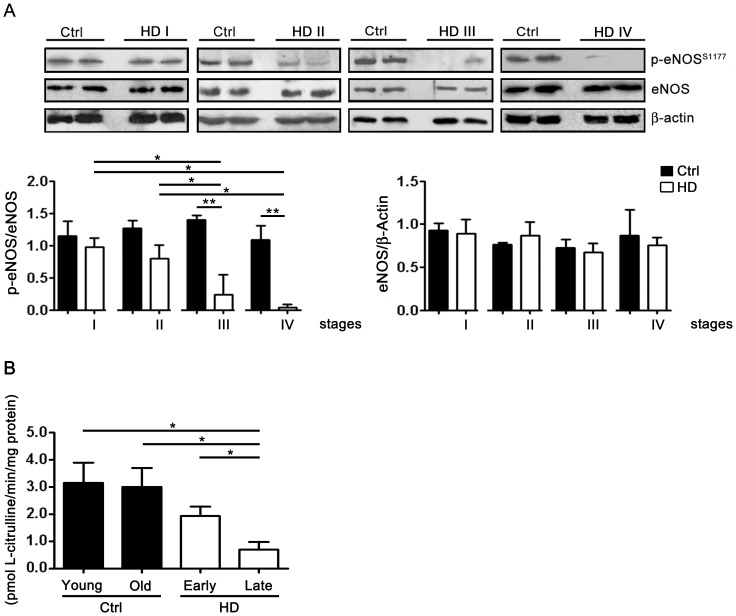
eNOS phosphorylation in platelets from HD patients and healthy controls. **(A)** Representative immunoblotting of eNOS phosphorylation at serine residue 1177 in platelets and densitometric analysis for p-eNOS^(1177)^ and eNOS (left) and for eNOS and β-actin (right). **(B)** Bar graph showing endothelial NOS activity in platelets from early (I-II) and late (III-IV) stage HD patients and healthy controls. Young Ctrl n = 5; Old Ctrl n = 4; Early stage n = 9; Late stage n = 9. Data are shown as mean ± SD. *, *p* <0.05. (One-way ANOVA followed by Tukey post test). F  = 8.766; df = 26.

To elucidate whether HD-derived platelet-mediated impaired vasorelaxation due to dysregulation of eNOS pathway, phosphorylation state at its serine residue 1177, was determined by biochemical studies. Levels of eNOS phosphorylation gradually decreased from stage II up stage IV HD patients in which phosphorylation signal was merely detectable (Figure 2A). eNOS phosphorylation in late stage HD patients was significantly reduced when compared with either stage I HD patients or control subjects. No significant difference was observed between early stage HD patients and healthy controls (Figure 2A). Conversely, eNOS expression did not change among all the groups (Figure 2A). In addition, no changes in the levels of β-actin were observed between HD patients and relative controls.

Furthermore, in order to clarify whether reduction of eNOS phosphorylation was associated with a defective eNOS activity along HD course, citrulline assay, the standard eNOS activity assay, was performed in platelets from different stated HD patients and healthy controls. Coherently with changes of eNOS phosphorylation, enzyme activity was found to be significantly decreased in patients with advanced HD compared to early HD patients and/or to controls subjects (Figure 2B).

### NO is reduced in platelets from late stage HD patients

**Figure 3:**
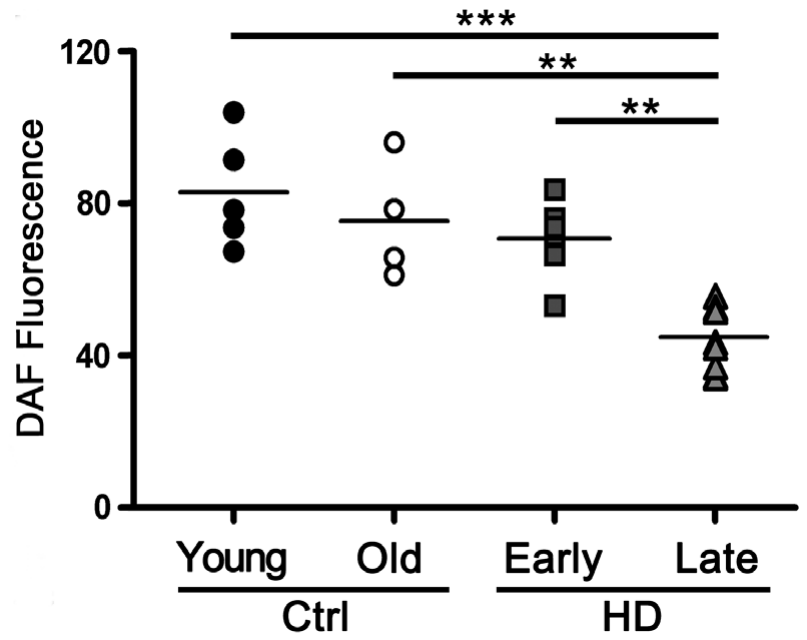
Production of NO in insulin-stimulated platelets from HD patients. Dot plot graph showing DAF fluorescence in platelets stimulated with insulin immediately after isolation from controls and HD patients. Lines indicate the mean values for each group. Each dot represents a single individual. Young Ctrl n = 5; Old Ctrl n = 4; Early HD n = 6; Late HD n = 6. *, *p* <0.05; **, *p* <0.001; ***, *p* <0.0001. (One-way ANOVA followed by Tukey post test). F = 14.52; df = 3.

To test whether impaired vasorelaxant response, evoked by HD-derived platelets might be attributable to abnormal NO production, platelet-derived NO from HD patients at different stage of the disease was quantitatively determined by DAF-FM DA. Consistent with decrease of both eNOS phosphorylation and activity (Figure 2A and B), NO release was markedly reduced in patients with advanced HD compared to early stage patients as well as to control subjects (Figure 3). No differences were found between early stage patients and control individuals (Figure 3).

### Discussion

Several studies highlighted peripheral abnormalities in HD and arise the hypothesis that the disease may be associated with a number of pathological changes outside the brain. A common finding across numerous studies is that peripheral cells (circulating blood lymphocytes, monocytes and platelets) represent good candidates for biochemical studies [5], [23], [24], as well as for searching potential peripheral biomarkers that correlate with the onset and/or the progression of the disease [6], [24].

It has been previously described an altered homeostasis of human platelets in HD [25], [26] and an increased density of Adenosine A2a receptor, a protein G-coupled receptor implicated in the regulation of different biochemical processes including the release of NO, in both CNS of transgenic HD mice [13], [27] and blood platelets of patients [5], [6]. However, whether platelets dysfunction contributes to HD pathogenesis, or is an epiphenomenon, remains unclear.

In this study we further examined the role of platelets in HD and postulate that the impairment of NO signaling in the peripheral district of patients might contribute to the pathology. In particular, we found that NO-mediated vasorelaxation, evoked by supernatant from HD-derived insulin-stimulated platelets was impaired in advanced HD. We also demonstrated stage-dependent changes of eNOS phosphorylation at serine residue (1177) associated with reduced eNOS activity and NO release in platelets from late stage HD patients. Importantly, as shown in Figure 1 and in Figure S1 in File S1, our results appear to be specifically related to HD pathology and not depending on aging process.

Interestingly, it has been previously shown that NO is produced in human platelets and that changes in intra-platelet NO production have important physiological and pathophysiological implications. The role of NO and its catalyzing enzyme, NOS, in neurodegenerative disease has been increasingly investigated over the past decade [27], [28]. However little is known about the role of NO in HD.

NO plays an important role in the modulation of the vascular tone. It exerts vasorelaxant action and favors antiplatelet effects limiting thrombotic events [29]. Impaired NO signaling in patients might support the high incidence of cardiovascular complications, which represent one of the leading causes of death in HD [30], [31].

Additionally, we observed that the progressive impairment of NO signaling along HD course seems not to depend exclusively on excessive oxidative stress. It has been previously reported that the effective half-life of NO and the vasorelaxation of aortic rings by NO is enhanced by a reduction in the concentration of superoxide radicals with superoxide dismutase (SOD) [32]. In our study, the administration of the antioxidant agent, TEMPOL, that mimics the action of SOD, did not rescue the altered vasorelaxation observed in the late stage HD patients. This result, therefore, allows us to suppose that superoxide radicals are not implicated in the impaired NO vascular action in patients with advanced HD. Interestingly, in support of this hypothesis, we found impaired activity of endothelial NOS enzyme in late stage HD patients.

Although the molecular mechanisms underlying the regulation of platelets eNOS activation and NO production are likely to be complex and will need to be further explored, we hypothesized that mHtt could affect eNOS phosphorylation through its interaction with a number of molecular targets including huntingtin-associated protein (HAP-1), CREB binding protein (CBP) and protein kinase B (AKT) [33], that have been previously described to interfere with eNOS signaling [28]. Reduction of eNOS expression and decreased NO levels in platelets from HD patients, could virtually reflect central defects and potentially clarify the molecular basis of cerebral hypoperfusion previously described in HD patients [34]. Although the clinical significance of altered eNOS/NO signal pathway is not yet elucidated, it is tempting to speculate that such impairment may be an important determinant that influences disease progression in HD.

In summary, our data indicate a gradual dysregulation of eNOS pathway in HD-derived platelets, which could represent a valuable tool for determining a possible link between peripheral district and CNS. To our knowledge this is the first biological evidence of such peripheral dysregulation in HD. We believe that changes of peripheral eNOS/NO pathway during HD course could represent a potential indicator of disease severity, useful to monitor the advanced HD stages of HD. The biological characterization of late stages of HD may open new insights to therapy of advanced, untreatable patients.

## Supporting Information

File S1 (DOCX)

## 
