# Supplementary material for: Nitric Oxide Dysregulation in Platelets from Patients with Advanced Huntington Disease
Source: PLoS One. 2014 Feb 25;9(2):e89745. doi: 10.1371/journal.pone.0089745 (PMC3934931; doi:10.1371/journal.pone.0089745)
Supplement: File S1 — (DOCX) [file pone.0089745.s001.docx]

**Nitric Oxide dysregulation in platelets from patients with advanced Huntington disease**

Albino Carrizzo^1#^, Alba Di Pardo^1#^, Vittorio Maglione^1^, Antonio Damato^1^, Enrico Amico^1^, Luigi Formisano^2^, Carmine Vecchione^1,3^*, Ferdinando Squitieri^1^*

^1^IRCCS Neuromed,  Pozzilli (IS), Italy; ^2^Department of Science and Technology, University of Sannio, Benevento, Italy; ^3^Department of Medicine and Surgery, University of Salerno, Salerno, Italy.

^#^ These authors contributed equally to this work.

* Co-corresponding authors:

Ferdinando Squitieri, M.D., Ph.D, e-mail: [ferdinando.squitieri@lirh.it](mailto:ferdinando.squitieri@lirh.it)

or

Carmine Vecchione M.D., e-mail: [cvecchione@unisa.it](mailto:cvecchione@unisa.it)

**Supporting Information**

**Legend to Supplementary Figure**

**Supplementary Figure 1.**

**(A)** Bar graph showing the statistical difference between the mean age of the two control groups (19 Young controls and 9 Old controls) (*p*<0.05 Student t-Test). (**B)** Dose–response curves of phenylephrine-precontracted aorta rings to supernatants derived from insulin-stimulated platelets from the two groups of control subjects (black line: dose-response curve in young controls; grey line: dose-response curve in old control subjects).

**
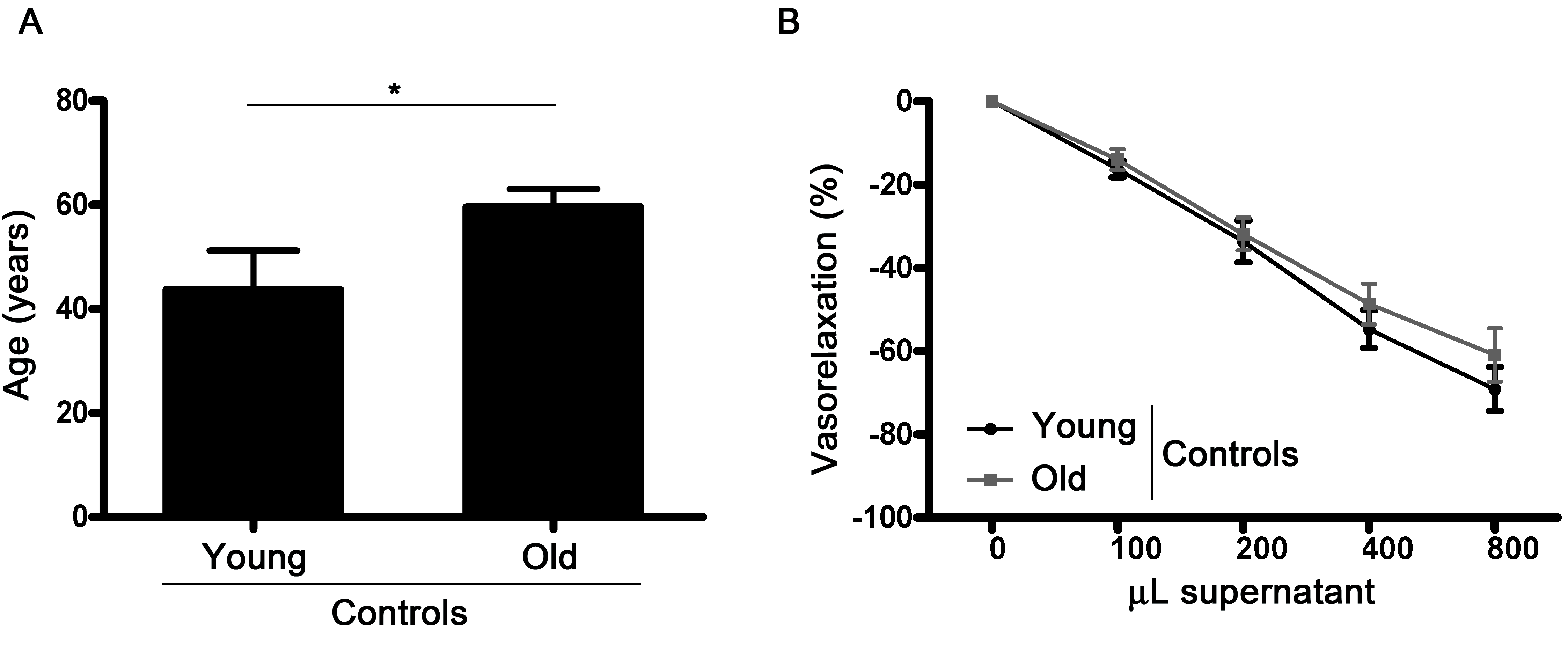
**

**Carrizzo et al. Figure S1.**
